# Global optimization of distillation columns using surrogate models

**DOI:** 10.1007/s42452-018-0008-9

**Published:** 2018-10-12

**Authors:** Tobias Keßler, Christian Kunde, Nick Mertens, Dennis Michaels, Achim Kienle

**Affiliations:** 1 Max Planck Institute for Dynamics of Complex Technical Systems, Sandtorstr. 1, 39106 Magdeburg, Germany; 2 Otto-von-Guericke-Universität Magdeburg, Universitätsplatz 2, 39106 Magdeburg, Germany; 3 TU Dortmund, Vogelpothsweg 87, 44227 Dortmund, Germany

**Keywords:** Global optimization, Distillation, Surrogate models, Kriging

## Abstract

Surrogate-based optimization of distillation columns using an iterative Kriging approach is investigated. Focus is on deterministic global optimization to avoid suboptimal local minima. The determination of optimal setups and operating conditions for ideal and non-ideal distillation columns, leading to mixed-integer nonlinear programming problems, serve as case studies. It is found that the optimization using the adapted Kriging approach yields similar results compared to the direct global optimization of the original problem in the ideal case, while it leads to a huge improvement compared to a multistart local optimization approach in the non-ideal case.

## Introduction

Rigorous optimization of distillation columns is of major interest in the chemical process industry due to its high economical impact. Due to the presence of discrete and continuous decision variables this leads to mixed-integer nonlinear programs (MINLP). Standard local optimization or stochastic optimization approaches can not guarantee that the optimum found by the optimizer is the global one. Alternatively, deterministic global optimization based on convex relaxations within a branch and bound framework has become an interesting approach for solving such problems, e.g., see the recent textbook by Locatelli and Schoen [6] for an introduction. However, computation times for distillation columns using standard model formulations from first principles are often extremely large [1, 7, 8].

To overcome this problem, different solution approaches were recently proposed. Quirante et al. [11] suggested to use surrogate models based on Kriging interpolation for optimization of distillation columns to reduce computational complexity. Main emphasis was on local optimization but it was also suggested to use Kriging models to reduce computational complexity in deterministic global optimization. Nallasivam et al. [8] presented an algorithm for calculating minimum energy requirements for thermally coupled distillation column configurations. The algorithm is based on a shortcut model which is only valid for ideal mixtures under minimum reflux conditions. An alternative approach for any reflux based on rigorous tray to tray models was proposed by Ballerstein et al. [1]. It applies to binary ideal mixtures. Illustration was demonstrated for a hybrid distillation crystallization process for isomer separation. The approach is based on monotonicity of the concentration variables in a binary distillation, which can be used to systematically reduce the search space. More recently this strategy could be extended in Mertens et al. [7] to ideal multicomponent distillation processes using a model reformulation strategy, which results in monotonicity of some aggregated concentration variables. However, an extension to non-ideal mixtures is in general not possible as will be argued in the present paper. Therefore, global optimization using Kriging models as proposed by Quirante et al. [11] is further investigated in some detail in this paper and compared to the previous approaches by Mertens et al. [7].

The outline of the paper is as follows: The general concept of Kriging interpolation is briefly explained in Sect. 2. Section 3 deals with ideal multicomponent distillation, which admits a rigorous global optimization using the reformulation by Mertens et al. [7]. It is shown that very similar results could be obtained with the Kriging approach using iterative refinement. Afterwards a highly non-ideal azeotropic mixture is considered in Sect. 4. Since rigorous global optimization is currently not possible with standard global optimization software within reasonable time, global optimization with the Kriging approach is compared with local optimization and thereby demonstrating the power of the global Kriging approach for highly non-ideal mixtures.

## Kriging models

Kriging models can be used to approximate complex mathematical models of real world processes. During the last years they gained increasingly more interest from engineers from different fields, such as chemical engineering, e.g. see [2] and [11]. The accuracy of Kriging models, as well as their complexity, depends on the number of reference points used for their generation. In this section the basic idea of an ordinary Kriging model is briefly sketched. The presentation mainly follows [2].

Given a vector-valued function $\varvec{f}:{\mathbb {R}}^{m}\rightarrow {\mathbb {R}}^{d}$ and a finite number of *reference points* $\bar{\varvec{x}}^{k}\in {\mathbb {R}}^m$, $k = 1,\dots , N$, a Kriging interpolation is a vector-valued function $\varvec{\hat{f}}:{\mathbb {R}}^m\rightarrow {\mathbb {R}}^d$ with $\varvec{\hat{f}}(\varvec{x}):= \varvec{q}(\varvec{x}) + \varvec{Z}(\varvec{x})$. Here, $\varvec{q}:{\mathbb {R}}^m\rightarrow {\mathbb {R}}^d$ is a vector-valued function consisting of polynomials, and $\varvec{Z}:{\mathbb {R}}^m\rightarrow {\mathbb {R}}^d$ is a vector-valued function used to describe the deviation of $\hat{\varvec{f}}(\varvec{x})$ from $\varvec{q}(\varvec{x})$. In ordinary Kriging models, as considered in this work, function $\varvec{q}(\varvec{x})$ is chosen to be a vector $\varvec{\xi }\in {\mathbb {R}}^d$ of suitable constants. Although this restriction seems to be rather strong, it does not affect the accuracy of the resulting surrogate model significantly for smooth functions, because most of the information is contained in $\varvec{Z}(\varvec{x})$ as noted by Papalambros and Wilde [9]. Function $\varvec{Z}(\varvec{x})$ is assumed to be a weighted sum of the deviations at all reference points with certain weights depending on $\varvec{x}$ that are defined by a weight function $\varvec{w}:{\mathbb {R}}^m\rightarrow {\mathbb {R}}^{N}$, i.e.

$$\begin{aligned} \varvec{Z}(\varvec{x}) = \sum _{k=1}^{N} w_k(\varvec{x})\cdot \big (\varvec{f}(\varvec{\bar{x}}^k)-\varvec{\xi }\big ). \end{aligned}$$

This leads to the following representation for $\hat{\varvec{f}}$.

$$\begin{aligned} \hat{\varvec{f}}^\top (\varvec{x}) = \varvec{\xi }^\top + \varvec{w}^\top (\varvec{x})\,\left( \begin{array}{c} \varvec{f}^\top (\varvec{\bar{x}}^{1}) - \varvec{\xi }^\top \\ \varvec{f}^\top (\varvec{\bar{x}}^{2}) - \varvec{\xi }^\top \\ \vdots \\ \varvec{f}^\top (\varvec{\bar{x}}^{N}) - \varvec{\xi }^\top \end{array} \right) . \end{aligned}$$

It is further postulated that vector $\hat{\varvec{f}}(\varvec{x})$ is identical to vector $\varvec{f}(\varvec{x})$ for each reference point, i.e.

1 $$\begin{aligned} \hat{\varvec{f}}(\varvec{\bar{x}}^k) = \varvec{f}(\varvec{\bar{x}}^k),\quad {\text {for all}} \quad k\in \{1,\dots ,N\}. \end{aligned}$$

In order to ensure the conditions in Eq. (1), it is requested that the weight function evaluated at the *k*th reference point coincides with the *k*th unit vector ${\mathbf {e}}_k\in {\mathbb {R}}^{N}$, i.e.

2 $$\begin{aligned} \varvec{w}(\varvec{\bar{x}}^k) = {\mathbf {e}}_k,\quad {\text {for all}}\quad k\in \{1,\ldots ,N\}. \end{aligned}$$

A parametrized function $c:{\mathbb {R}}^m\times {\mathbb {R}}^m\rightarrow {\mathbb {R}}$ and a matrix $\varvec{R}$ are further used to calculate the weights $\varvec{w}(\varvec{x})$ as follows.

$$\begin{aligned} \varvec{w}^\top (\varvec{x}) = \left( c(\varvec{x},\varvec{\bar{x}}^{1}), \dots , c(\varvec{x},\varvec{\bar{x}}^{N}) \right) \, \varvec{R} \end{aligned}$$

Due to (2), matrix $\varvec{R}$ needs to be

3 $$\begin{aligned} \varvec{R} = \left( \begin{array}{ccc} c(\varvec{\bar{x}}^{1},\varvec{\bar{x}}^{1}) &{} \dots &{} c(\varvec{\bar{x}}^{1},\varvec{\bar{x}}^{N}) \\ \vdots &{} \ddots &{} \vdots \\ c(\varvec{\bar{x}}^{N},\varvec{\bar{x}}^{1}) &{} \dots &{} c(\varvec{\bar{x}}^{N},\varvec{\bar{x}}^{N}) \end{array} \right) ^{-1} . \end{aligned}$$

Note that function *c* and the reference points must be chosen such that matrix $\varvec{R}$ is invertible. In the literature, various strategies for finding a suitable function *c* and appropriate reference points are available, e.g. see [5].

In case of $\varvec{f}$ being a Gaussian process, minimizing the variance of the estimation error $\hat{\varvec{f}}(\varvec{x})-\varvec{f}(\varvec{x})$ leads to function values $c(\varvec{x}^1,\varvec{x}^2)$ being the covariance between the points $\varvec{x}^1$ and $\varvec{x}^2$. When interpolating a deterministic function (as considered here), any function $c(\varvec{x}^1,\varvec{x}^2)$ may be feasible and the best choice is non-trivial. The choice of the function determines how the model fits the data. Therefore, it usually contains parameters that have to be optimized in order to generate a surrogate model with a good fit. The most commonly used function is an exponential function of the following form [2]

$$\begin{aligned} c(\varvec{x}^{1},\varvec{x}^{2}) = \exp \left( -\sum ^m_{i=1} \theta _i \Vert \varvec{x}_{i}^1 - \varvec{x}_{i}^2 \Vert ^{p_i}\right) . \end{aligned}$$

For $\varvec{x}^1 = \varvec{x}^2$, value $c(\varvec{x}^1,\varvec{x}^2)$ equals one, while it tends to zero when the distance between the points $\varvec{x}^1$ and $\varvec{x}^2$ increases. For every component *i*, parameter $\theta _i$ defines the speed of this tendency and parameter $p_i$ denotes the smoothness of *c*. In summary, $\varvec{Z}(\varvec{x})$ is calculated as

$$\begin{aligned} \varvec{Z}^\top (\varvec{x}) = \left( c(\varvec{x},\varvec{\bar{x}}^{1}), \dots , c(\varvec{x},\varvec{\bar{x}}^{N}) \right) \cdot \varvec{F}, \end{aligned}$$

with constant matrix $\varvec{F}$ given by

$$\begin{aligned} \varvec{F} = \varvec{R}\cdot \left( \begin{array}{c} \varvec{f}^\top (\varvec{\bar{x}}^{1}) - \varvec{\xi }^\top \\ \varvec{f}^\top (\varvec{\bar{x}}^{2}) - \varvec{\xi }^\top \\ \vdots \\ \varvec{f}^\top (\varvec{\bar{x}}^{N}) - \varvec{\xi }^\top \end{array} \right) . \end{aligned}$$

Thus, there are some unknown parameters that have to be calculated, namely $\varvec{\xi }$, $\theta _i$ and $p_i$. Following [11], values of the parameters are obtained by maximizing the logarithmic likelihood function

$$\begin{aligned} \log \left( L\right) =&-\,N/2 \left( \log \left( \sigma ^2\right) +\log \left( 2 \pi \right) \right) \\&- \,1/2 \log \left( \Vert \varvec{R}\Vert \right) \\&-\, 1/\left( 2 \sigma ^2\right) \left( \varvec{Y}\left( \varvec{\bar{x}} \right) - \varvec{1}\varvec{\xi }\right) ^\top \varvec{R}^{-1}\left( \varvec{Y}\left( \varvec{\bar{x}} \right) -\varvec{1}\varvec{\xi }\right) , \end{aligned}$$

with

$$\begin{aligned} \varvec{\xi }&= \left( \varvec{1}^\top \varvec{R}^{-1}\varvec{1} \right) ^{-1}\left( \varvec{1}^\top \varvec{R}^{-1}\varvec{Y}\left( \varvec{\bar{x}} \right) \right) ,\\ \sigma ^2&= 1/N \left( \varvec{Y}\left( \varvec{\bar{x}}\right) - \varvec{1}\varvec{\xi }\right) ^\top \varvec{R}^{-1} \left( \varvec{Y}\left( \varvec{\bar{x}}\right) - \varvec{1}\varvec{\xi } \right) ,\\ \varvec{Y}\left( \varvec{\bar{x}}\right)&= \left( \begin{array}{ccc} \varvec{f}_1(\varvec{\bar{x}}^{1}) &{} \dots &{} \varvec{f}_d(\varvec{\bar{x}}^{1}) \\ \vdots &{} \ddots &{} \vdots \\ \varvec{f}_1(\varvec{\bar{x}}^{N}) &{} \dots &{} \varvec{f}_d(\varvec{\bar{x}}^{N}) \\ \end{array} \right) . \end{aligned}$$

In this work the MATLAB function “fmincon” is used for the identification of the parameters, i.e. for the fitting of the Kriging models. Pseudo code describing the fitting procedure is shown in Algorithm 1.

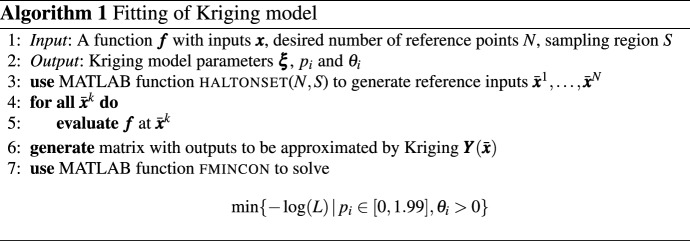



**Fig. 1:**
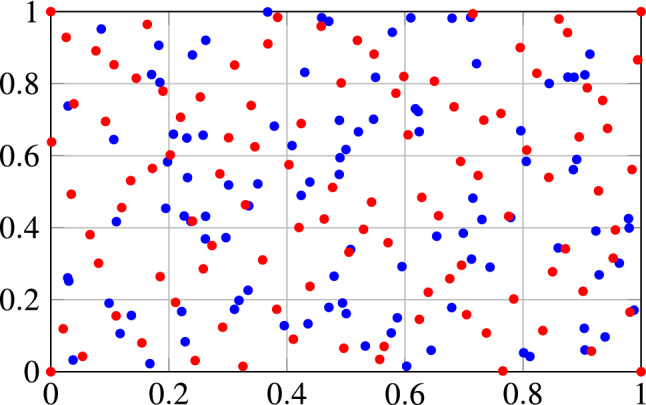
Difference between Halton sequence (red dots) and random number sequence (blue dots) with 100 samples each

As the choice of the reference points may greatly influence the accuracy, it is important to use a space filling approach instead of using randomly generated data points. Otherwise clustering in unimportant regions of the model may occur and can result in a surrogate model of poor quality. In this work, a *Halton sequence* [4] is used to cover the space evenly. Figure 1 shows an illustrative example of the difference between a Halton sequence, depicted as red dots, and a random number sequence, depicted as blue dots, with 100 samples each. While the red dots cover the space evenly, clustering in the blue dots occurs, e.g. in the upper right corner. The integer variables are enumerated through the sampling procedure.

After a first Kriging model is generated, it is optimized using the deterministic global optimization software BARON [12]. Following [2], a second, refined Kriging model is constructed by restricting the sampling region to a certain neighborhood around the found solution of the first Kriging model. The second Kriging model is likely to be more precise in the region of interest. It may happen that one of the variables in the solution of the second Kriging model attains its value at the boundary of its respective domain. In this case, a further Kriging model is generated using a neighborhood around the optimal solution of the second Kriging model as sampling region. Note that the latter neighborhood also covers regions that are not contained in the neighborhood around the optimal solution of the first Kriging model.

This refinement approach is related to trust region methods that are used as a numerical solving strategy to compute locally optimal solutions for non-linear optimization problems. Trust region methods are based on an iterative procedure in that an approximation model of the original problem is solved in each step. In each iteration the corresponding approximation model is restricted to a certain sub-region usually containing the solution of the previous iteration. The size of the sub-regions may depend on the assumed model quality estimated with information from previous steps. We refer to the work [13] for a recent survey on trust region methods.

All Kriging models constructed through our computations are implemented as MINLPs and solved using the GAMS 24.6.1 framework with the deterministic global optimization software BARON 15.9.22., Cplex 12.6.3 is used as LP/MIP subsolver and CONOPT 3.17A is utilized as NLP subsolver. The calculations are carried out on a Linux PC with 3.40 GHz Intel Core i7-6700 CPU and 16 GB memory.

## Ideal distillation

**Fig. 2:**
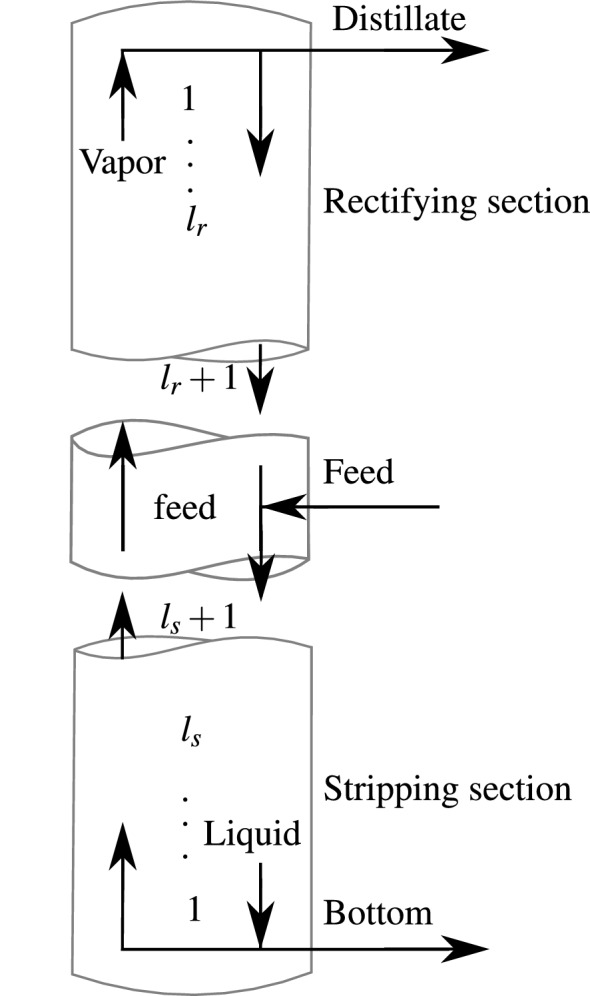
Distillation column scheme with variable number of stages in the upper and lower part of the column and variable feed location. The number of stages is $l_r+l_s+1$, the feed location is at stage $l_s+1$

As a first case study an ideal four-component distillation column with variable number of stages and a variable feed stage is chosen. A sketch of such a distillation column is depicted in Fig. 2. The distillation process aims to separate the two more volatile components from the two less volatile components under given purity requirements, while minimizing the total annualized cost (TAC) of the process. The mathematical model description as well as the numerical test instances are taken from Mertens et al. [7]. Despite the fact that the vapor-liquid-equilibrium (VLE) of the column is modeled as ideal, i.e. with constant relative volatilities, finding a global optimum of the corresponding MINLPs is rather challenging using standard model formulations. It was, however, demonstrated in Mertens et al. [7] that a model reformulation in combination with tailor-made optimization techniques can reduce the computational effort considerably. Thus, globally optimal solutions for different test instances are available and can be used as references to investigate the accuracy of the surrogate based approach presented in this work.

The case study deals with three different mixtures that are separated with three different product specifications each. This leads to a total number of nine test instances. The mixtures are labeled by numbers 1, 2 and 3 where the difficulty of the separation task increases from mixture 1–3. The product specifications are given by the purity requirements on the distillate, i.e. on the mixture consisting of the two more volatile components, and on the bottom product, i.e. the mixture consisting of the two less volatile components. Different product specifications are labeled by letters *a, b* and *c*, and become more restrictive for the separation task from *a* to *c*. The concrete parameter setting defining each test instance is provided in Mertens et al. [7].

Note that feasible solutions to the considered MINLPs represent feasible distillation column designs of the corresponding separation tasks. A characterization of each such designs is given by the length $l_r$ of the rectifying section, i.e. the part above the feed stage (see Fig. 2), by the length $l_s$ of the stripping section, i.e. the part below the feed stage, by the distillate flow rate *D* in mol/s and by the vapor flow rate *V* in mol/s. For the known optimal solutions to our test instances, these characteristic properties are summarized in Table 1. The computation times needed to carry out the optimization using a SCIP optimization framework are given in column “time” in seconds.

Algorithm 1 described in Sect. 2 is applied to a model of the distillation column implemented in MATLAB, where the input variables $\varvec{x}\in S$ are $V\in [0.85,3]$, $D\in [0.8,1]$ and $l_r,l_s\in [1,24]$ and the desired output variables $\varvec{Y}(\varvec{x})$ are the concentrations of the product mixtures at the top and the bottom of the column. The resulting Kriging models are implemented and optimized in GAMS/BARON. The objective function is taken from Mertens et al. [7] and the purity requirements are implemented as inequality constraints. The results obtained for all test cases can be found in Table 2. The results for the cases labeled with subscript 1 refer to the results obtained with the first Kriging model, generated with 696 sampling points. Results for the cases labeled with subscript 2 are obtained by the second Kriging models generated with the iterative sampling approach presented in Sect. 2, using 1156 sampling points. The new sampling region for the degrees of freedom lies within a 25% range of the found optimum for the continuous variables, *V* and *D*, and $\pm \, 2$ around the optimum of the integer variables, $l_s$ and $l_r$. It can be seen that the iterative Kriging optimization approach is able to calculate a solution lying in the neighborhood of an actual global optimum. However, it is important to note that the iterative Kriging approach may lead to solutions that are not close to a global optimal one. This may be due to a possibly high inaccuracy of the first Kriging model that may yield initial solutions that are already far away from a global optimum. If the first Kriging model is then refined around a solution of poor quality, it may not be possible to arrive at an actual global optimum as it is no longer captured by the refined sampling region of the subsequent Kriging model.

**Table 1:** Computational results from reference calculations [7] with a feed flow of 1.8 mol/s

Case	TAC	*D*	*V*	$l_s$	$l_r$	Time
1a	23566	0.9	0.9408	3	1	134
1b	33597	0.9	1.2796	4	3	2097
1c	41177	0.9	1.4034	6	6	1211
2a	25419	0.9	0.99	4	1	349
2b	37109	0.9	1.3519	5	4	939
2c	46727	0.9	1.5416	7	7	2512
3a	27993	0.9	1.0719	4	2	386
3b	42633	0.9	1.499	6	5	3541
3c	55629	0.9	1.7518	9	8	9746

Note that in most cases the first solutions obtained do not meet the constraints due to the inaccuracy of the first Kriging model. However, after the refinement of the sampling region the constraints are met by the found optimal solutions.

Strict comparison of computation times of the different approaches is not possible due to different hardware and optimization software configurations. However, it was observed that the reference model typically could not be solved within 10 h, whereas the reformulated model with some tailor made bound tightening strategies was most of the time solved in less than an hour. For the detailed statistics we refer to Mertens et al. [7]. The computation times needed for the optimization using the Kriging approach presented with standard software (BARON) is given in Table 2 in the column “time” in seconds. Additional time is needed for the sampling (around 45 s) and the fitting of the Kriging model (around 680 s). The computation times of both approaches lie in the same order of magnitude.

## Non-ideal distillation

In the previous section focus was on separation of ideal mixtures with constant relative volatilities. Next, a highly non-ideal azeotropic mixture with variable volatilities is considered.

From Sect. 3 it is clear that the global optimization of the reformulated model is preferable to the optimization of the Kriging model, because the global optimality of the result obtained with the Kriging approach can not be guaranteed. However, global optimization of the rigorous model is computationally even much more expensive compared to the previous example. Further, a monotonic reformulation $\hat{x_i}(z)$ of the molar fractions $x_i(z)$ like in the previous ideal case is also not possible anymore, as will be shown in the following.

**Table 2:** Computational results using surrogate model. Values with a subscript higher than 1 are calculated using an adaptive sampling technique

Case	TAC	*D*	*V*	$l_s$	$l_r$	Time
$1{\hbox {a}}^{\text {krig}}_1$	23466	0.9045	0.9045	3	2	244
$1{\hbox {a}}^{\text {krig}}_2$	24318 ($+$ 3.19%)	0.9001 ($+$ 0.01%)	0.9424 ($+$ 0.17%)	3	2 (+1)	16
$1{\hbox {b}}^{\text {krig}}_1$	37002	0.8992	1.3104	6	4	2053
$1{\hbox {b}}^{\text {krig}}_2$	34563 ($+$ 2.88%)	0.8998 ($-$ 0.02%)	1.2831 ($+$ 0.27%)	4	4 (+1)	105
$1{\hbox {c}}^{\text {krig}}_1$	59179	0.8977	2.0610	5	8	4885
$1{\hbox {c}}^{\text {krig}}_2$	44362 ($+$ 7.73%)	0.9006 ($+$ 0.07%)	1.5281 ($+$ 8.89%)	5 ($-$ 1)	7 (+1)	179
$2{\hbox {a}}^{\text {krig}}_1$	27280	0.9787	0.9787	5	3	278
$2{\hbox {a}}^{\text {krig}}_2$	26216 ($+$ 3.14%)	0.9005 ($+$ 0.06%)	0.9942 ($+$0.42%)	4	2 (+1)	38
$2{\hbox {b}}^{\text {krig}}_1$	38383	0.8963	1.3296	6	5	703
$2{\hbox {b}}^{\text {krig}}_2$	37992 ($+$2.38%)	0.8998 ($-$ 0.02%)	1.3505 ($-$ 0.10%)	5	5 ($+$ 1)	122
$2{\hbox {c}}^{\text {krig}}_1$	58178	0.9024	2.022	7	6	6202
$2{\hbox {c}}^{\text {krig}}_2$	50639 ($+$ 8.38%)	0.9000	1.731 ($+$ 12.29%)	5 ($-$ 2)	8 ($+$ 1)	104
$3{\hbox {a}}^{\text {krig}}_1$	30142	0.8979	1.0642	5	4	312
$3{\hbox {a}}^{\text {krig}}_2$	28785 ($+$2.83%)	0.9012 ($+$ 0.13%)	1.1067 ($+$ 3.25%)	4	2	182
$3{\hbox {b}}^{\text {krig}}_1$	44871	0.8988	1.5884	7	4	1151
$3{\hbox {b}}^{\text {krig}}_2$	43769 ($+$ 2.66%)	0.9003 ($+$ 0.03%)	1.5445 ($+$ 3.04%)	6	5	107
$3{\hbox {c}}^{\text {krig}}_1$	59683	0.9011	1.9854	8	7	2122
$3{\hbox {c}}^{\text {krig}}_2$	56986 ($+$ 2.44%)	0.9001 ($+$ 0.01%)	1.7220 ($-$ 1.70%)	10 (+1)	9 (+1)	200

The percentage difference of the results w.r.t. the known globally optimal solutions is given in brackets

Take the transformed states $\hat{x_i}$ as functions of the molar fractions $x_i$

4 $$\begin{aligned} \hat{x}_1&= x_1, \end{aligned}$$

5 $$\begin{aligned} \hat{x}_2&= x_1 + x_2,\end{aligned}$$

6 $$\begin{aligned} \hat{x}_3&= 1, \end{aligned}$$

and their derivatives with respect to the spatial coordinate *z*

7 $$\begin{aligned} \dfrac{\partial \hat{x}_1}{\partial {\mathrm {z}}}&= \dfrac{\partial x_1}{\partial {\mathrm {z}}} \ge 0, \end{aligned}$$

8 $$\begin{aligned} \dfrac{\partial \hat{x}_2}{\partial {\mathrm {z}}}&= \dfrac{\partial (x_1 + x_2)}{\partial {\mathrm {z}}} \end{aligned}$$

9 $$\begin{aligned}&= \dfrac{\partial (x_1 + 1 - x_3 - x_1)}{\partial {\mathrm {z}}}\end{aligned}$$

10 $$\begin{aligned}&= - \dfrac{\partial x_3}{\partial {\mathrm {z}}} \ge 0, \end{aligned}$$

11 $$\begin{aligned} \dfrac{\partial \hat{x}_3}{\partial {\mathrm {z}}}&= 0, \end{aligned}$$

for the proof.

The assumption of positive derivatives in (7) and (10) always holds for the distillation of ideal mixtures, but it cannot be guaranteed for non-ideal mixtures because of the occuring azeotropes.

**Fig. 3:**
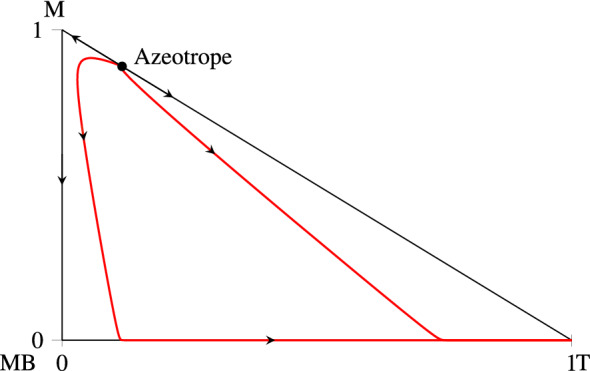
Residue curve map of the mixture Methanol (M)–Methylbutyrate (MB)–Toluene (T). Arrows indicate the phase behavior for increasing temperature, i.e. from the top to the bottom of the column

One example for such a mixture is the distillation of Toluene, Methanol and Methylbutyrate [3], which is investigated here. It is easy to see from the residue curve map, representing the column profiles at total reboil and reflux, depicted in Fig. 3, that the above condition that the column profiles have to be monotonic for at least two of the components is not met.

To model non-ideal phase behavior, several activity coefficient models, such as UNIQUAC and Wilson [10], may be used. In the present case study a Wilson approach is chosen. The column is assumed to be operated at a constant pressure of 1.013 hPa, the feed rate *f* is set to 1.8 mol/s and the feed composition mole fraction $x_{\mathrm {Feed}}$ is [0.2806, 0.6566, 0.0628], where the first entry refers to Toluene, the second entry to Methanol and the third entry to Methylbutyrate. The aim of the optimization is to find optimal operating conditions such that at least 80 % of component Toluene contained in the initial feed flow rate is gained at the bottom of the column with a purity of at least 95 %. This gives rise to the two constraints

12a $$\begin{aligned} x_{\mathrm {T,Bott}}&\ge 0.95, \end{aligned}$$

12b $$\begin{aligned} x_{\mathrm {T,Bott}}\cdot B&\ge 0.8 \cdot f \cdot x_{\mathrm {Toluene,Feed}}=0.4041, \end{aligned}$$

where *B* is the bottom flow rate in mol/s. The same objective function as in the previous example is used. The degrees of freedom are the distillate flow rate $D\in [1.1, 1.7]$ mol/s, the vapor flow rate $V\in [2,13]$ mol/s, as well as the number of stages $l_r+l_s+1$ and the feed location $l_s+1$ with $l_r,l_s\in [1,24]$.

Since global optimization with the rigorous model or its reformulation like in the ideal case is not possible within reasonable time anymore, local optimization is applied as a reference. For this, the local multi-start heuristic provided by BARON is applied with 5000 starting points. The results are displayed in the second row of Table 3 labeled with “Local”.

**Table 3:** Computational results for non-ideal distillation. Cases with numbered a subscript higher than 1 are calculated using adaptive sampling techniques, cases labeled with M are calculated using MATLAB

Case	TAC	*D*	*V*	$l_s$	$l_r$	Time
Local	134,373	1.3747	4.2063	4	18	8873
${\hbox {Surrogate}}_1$	71,759	1.3772	2	8	17	3527
${\hbox {Surrogate}}_2$	103,355	1.3747	2.7036	11	20	446
${\hbox {Surrogate}}_3$	94,255	1.3747	2.4403	13	18	362
Local$_{\mathrm {M1}}$	93,189	1.3747	2.4095	13	18	
Local$_{\mathrm {M2}}$	86,604	1.3747	2.3380	17	11	

**Fig. 4:**
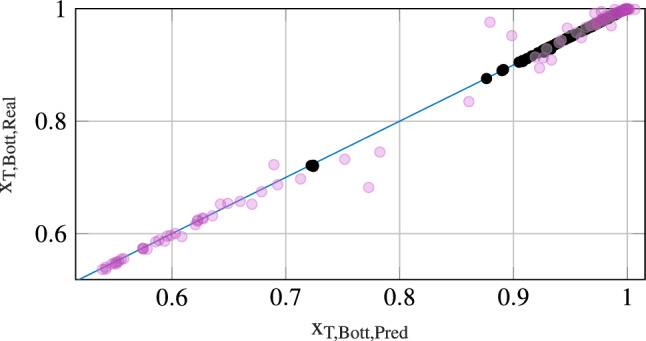
Cross validation of non-ideal distillation column in terms of $x_{T,Bott}$, the mole fraction of Toluene in the Bottom product. The magenta dots show the results for the first Kriging model, the black dots show the results for the second Kriging model

The Kriging models have been fitted using 1190 sampling points. A cross validation of the first Kriging model (case “Surrogate$_1$” in Table 3) was done with 100 points and is depicted in Fig. 4 as magenta dots. The first Kriging model is rather inaccurate, especially in the occuring discontinuity of $x_{\mathrm {T,Bott}}$, that is roughly contained in the interval (0.72, 0.85). This is due to the large sampling region and due to the complexity of the problem. As a result, the operating conditions obtained in the first optimization are violating the constraints with $x_{\mathrm {T,Bott}}=0.9034$ and $x_{\mathrm {T,Bott}}\cdot B =0.3820$ mol/s.

Since the operating conditions found during the reference optimization “Local” are far away from the optimal operating conditions for “Surrogate$_1$”, the sampling region for the adaptive Kriging approach is chosen to be larger than in the ideal case, with $\pm \,3$ for the integer variables, $\pm \,25\,\%$ for *D* and $+3$ for *V*. After sampling around the obtained optimum and generating the second Kriging model (“Surrogate$_2$” in Table 3) a second cross validation with 100 points was done. The results are shown in Fig. 4 as black dots. The second Kriging model is much more accurate and is able to model the discontinuity quite well. It turns out, that the new optimum obtained for Surrogate$_2$ satisfies the desired conditions stated in Eqs. (12).

Note that the values for $l_r$ and $l_s$ given by the computed optimal solution of the second Kriging model lie on the boundary of their respective sampling region. Hence, a third Kriging model (Surrogate$_3$) is generated around this optimum with $l_r,l_s\pm \,3$, *D* within a 25 % range and $V\in [2,4]$. In the third optimization the objective value could be lowered further. Comparing the result of the local optimization with the iterative global optimization of the Kriging models, the objective value is finally lowered by $29.86\%$.

The purity of Toluene achieved with the operating conditions of “Surrogate$_3$” is higher than the required specification of 95%. To decrease the objective function value further, a local search using the obtained operating conditions as initial conditions is done in MATLAB using the high-fidelity reference model and thereby reducing the purity to 95%. The results of that optimization can be found in Table 3 (see row “Local$_{\mathrm {M1}}$”).

Based on the solution “Local$_{\mathrm {M1}}$” and combining expert knowledge with further local optimization iterations the solution that is shown in row “Local$_{\mathrm {M2}}$” of Table 3 can be obtained, which is the best local optimum we found through our computations. However, the improvement achieved by applying “Local$_{\mathrm {M2}}$” compared to the use of “Surrogate$_3$” is minor with respect to the improvement that is achieved by applying “Surrogate$_3$” compared to the use of “Local”.

The computation times for each optimization are given in seconds in the column “time” of Table 3.

## Conclusion

In this work, two case studies concerning the distillation of multi-component mixtures have been conducted, where global optimization techniques have been applied to surrogate models of the distillation columns investigated.

It was shown that the reformulation developed earlier by the authors is not always applicable in the case of a distillation of a mixture with non-ideal VLE, rendering the problems unsolvable within a reasonable amount of time for standard deterministic global optimization software. In these cases, the iterative global optimization of surrogate models is a good alternative, which yields better optima than ordinary local solver and in some cases comes close to the global optimum. It can, however, not be guaranteed that the solution obtained by the optimization of these surrogate models is the actual global optimum or lies in a close neighborhood of it.

Further work will be concerned with more advanced adaptive sampling methods and algorithms for the generation of global models.
